# The American Hospital Association

**Published:** 1916-10-21

**Authors:** 


### THE AMERICAN HOSPITAL ASSOCIATION.

### ITS PROGRESS AND PRESENT POSITION.

The Eighteenth Annual Conference of this Association was held at Philadelphia from September 26 to 29 inclusive. It was one of the most largely attended of these Conferences, and the additions to the membership arising out of it were larger than those in any previous year. The Treasurer reported that the Association would commence the new year with a balance of upward of eight hundred pounds in hand. The President of the Conference was Dr. Winford H. Smith, Superintendent of the Johns Hopkins Hospital, Baltimore, Maryland, who discharged his duties with admirable expedition, judicial impartiality, and mastery of the subjects dealt with.

The Philadelphia Conference differed from those we have previously attended in the circumstances that the programme each day was so full as to render it practically impossible to permit, much less to encourage, the full discussion of many important matters raised by the papers and addresses. These subjects included a most excellent, courageous, and masterly address by Dr. Winford H. Smith, which we hope to publish next week in full. Although the address is written from the American point of view, many of the topics have an interest for hospital workers throughout the world, and the criticisms and suggestions it contains deal with several full and complete subjects. One of the most successful matters dealt with by the Conference was the report of a committee on the constitution and by-laws, presented by Mr. Richard P. Borden, Trustee of the Union Hospital, Fall River, Mass., who through successive meetings exhibited such a mastery of the points raised and such skill in exposition that he succeeded, with the co-operation of the President of the Association, Dr. Howard, Dr. Hurd, and other leaders, in removing all opposition and carrying both the constitution and the by-laws through the Conference with the unanimous consent of all present. The new constitution brings the working machinery well up to date, and may form a model for similar organisations in other countries.

### The Best of the Papers.

The general work of the Conference was divided into two sections, the greater section including all branches of hospital work and treatment, whilst the smaller section was confined to community and small hospitals and subjects connected with them. The most interesting papers in the latter section related to (*a*) "How may a Hospital Superintendent Promote more Scientific Work in a Small Hospital?" and (*b*) "How are the Superintendents in Small Hospitals to be Trained?" We hope to have an opportunity to deal with these matters at length on a future occasion.

Amongst the most important papers in the main section was one by Dr. W. L. Babcock, Superintendent of Grace Hospital, Detroit, Michigan, entitled "The Open-Door Hospital"—that is, a hospital which admits every practitioner resident within the district which it may serve, subject only to the decision of the trustees and superintendent as to the admission to the hospital of a practitioner whose repute may be unsatisfactory. Personally, we believe the open-door hospital to be based upon a plan ill-calcalulated to produce administrative efficiency and the best results. This system has not answered in the smaller English hospitals, and though reputed to be favoured by politicians in the United States, we have reason to doubt the average working efficiency of this system even there.

A most interesting and instructive paper on "What Dispensary Work should Stand For" was read by Dr. Richard C. Cabot, of Boston, Mass. Mr. Michael M. Davis, jun., Chairman of the Boston Dispensary, gave a most interesting account of its work, illustrated by limelight pictures. We are of opinion that the establishment of dispensaries in England of the type of that described by Dr. Cabot on a consultative basis would have a strengthening influence upon the working of the National Insurance Acts so far as medical treatment and its results are concerned.

Another paper, which we hope to publish in full, is that on the "Hospital Dietary," by Dr. Elliott P. Joslyn, Associate Professor of Medicine, Harvard University. It was a most useful and practical statement, full of wise suggestions, upon a subject which requires more scientific treatment in every hospital than it at present receives in this country.

The meetings on Thursday, September 28, were, in the larger section, of surpassing interest and importance. They included a very courageous and earnestly presented treatise upon the hospitals for the purpose of arriving at higher standards of practice. Mr. Bowman made a great impression upon the large audience present in his "Study of Hospitals for the Purpose of Arriving at Proper Standards." He offered the whole-hearted co-operation of the American College of Surgeons with the American Hospital Association with a view to remedy the existing abuses and greatly strengthen the hospitals.

Dr. A. R. Warner, Superintendent of Lakeside Hospital, Cleveland, Ohio, read a strong report of "The Committee on Hospital Finances and Costs Accounting." This paper revealed the absence of uniformity in the system of hospital accounting in the United States, and resulted in the appointment of a Committee of Three to deal with the important points raised, with power to report.

Mr. Bartine, Superintendent of the Hospital for Ruptured and Crippled Children, New York City, read a paper on "Building the Hospital," in which he expounded his views in regard to departments and rooms. A striking paper was that by Dr. George O'Hanlon, Superintendent of Bellevue and Allied

Hospitals, New York City, being a report of a Committee on Efficiency and Progress. It was a very useful document compiled with ability upon a plan which facilitated the understanding of its aims and objects.

Probably the ablest single paper was that by Dr. S. S. Goldwater, Director of the Mount Sinai Hospital, New York City, entitled the "Hospital and the Surgeon." This paper covered the following points, each of which was expanded in detail with much vigour and convincing thoroughness. They included: Making the Surgeon, Selecting the Surgeon, Paying the Surgeon, Aiding the Surgeon, Regulating the Surgeon, and the Surgical Monopoly. The first section of this paper is given in full in the *Modern Hospital* for October 1916, pages 273 to 277 inclusive. Each section of the subject as enumerated was dealt with with convincing directness and judicial fairness. It is full of points well worthy of the trustees, committees, and superintendents of all hospitals of importance, and will exercise, we hope, widespread influence for good upon the *personnel* and working of the surgical department of many hospitals. All hospitals would be better off if those responsible for the discipline and efficiency of every great medical institution were to obtain this paper and to read it critically and carefully. We propose to publish it shortly.

Few papers more instructive or convincing have ever been presented to a hospital conference than that on "Convalescent Hospitals, Methods, and Results," by Dr. Frederick Brush, Superintendent of Burke Foundation, White Plains, New York. Dr. Brush gave up the Medical Superintendency of a large hospital in order to take up the important questions involved in the reorganisation and modernisation of convalescent institutions. He has aimed at the maximum of effective results by methods and treatment involving close observation and continuous oversight on the part of those immediately responsible for the comfort, treatment, and general welfare of the patients.

A Suggestion for the Future.

Many of the papers presented to the Conference were excellent in quality and of real practical value. The last two days of the programme resulted in holding the undivided interest of crowded assemblies of hospital workers in active control of the principal hospitals in the United States and Canada. They contained evidence of the splendid spirit of research for knowledge and improved conditions which possessed the younger superintendents especially. We have no doubt that many of these gentlemen could have given valuable information had the arrangements permitted a full discussion and the encouragement of speakers. Nothing could more clearly demonstrate the justice of this conclusion than the fact that a large number of hospital superintendents remained interested in the proceedings for something like twelve continuous hours, from 10 a.m. until after 11 p.m. on Thursday, September 28.

We hope that the educational influence of the American Hospital Association will have so important a bearing upon the general efficiency of hospitals in the United States that under the controlling hand of the new constitution and the direction of the new officers it may be found practical in future to select the best and most informative of the papers submitted, and to place one of them at the head of the list of each successive meeting during the Conference in 1917. By such an arrangement, with ample time given for discussion and encouragement to the younger superintendents especially to show their qualifications and efficiency by taking part in practical debate, results should follow which could not fail to influence for good many hospitals throughout the countries represented at each year's Conference.

